# Molecular epidemiology and characteristics of respiratory syncytial virus among hospitalized children in Guangzhou, China

**DOI:** 10.1186/s12985-023-02227-4

**Published:** 2023-11-22

**Authors:** Sajid Umar, Rongyuan Yang, Xinye Wang, Yuntao Liu, Peifeng Ke, Sheng Qin

**Affiliations:** 1 Global Health Research Center, Duke Kunshan University, Kunshan, China; 2 Division of Natural and Applied Sciences (DNAS), Duke Kunshan University, Kunshan, China; 3 Key Laboratory for Infectious Disease, The Second Affiliated Hospital of Guangzhou University of Chinese Medicine, Guangzhou, China; 4 School of Biomedical Sciences, Faculty of Medicine, University of New South Wales, Sydney, NSW Australia; 5 Emergency Department, The Second Affiliated Hospital of Guangzhou University of Chinese Medicine, Guangzhou, China; 6 Department of Laboratory Medicine, The Second Affiliated Hospital of Guangzhou University of Chinese Medicine, No. 111 Dade Road, Yuexiu District, Guangzhou, China

**Keywords:** Respiratory syncytial virus (RSV), Molecular epidemiology, Genetic diversity, Mutations, Phylogenetic analysis, Evolution, Guangzhou, China

## Abstract

**Background:**

Human respiratory syncytial virus (RSV) is a leading cause of acute lower respiratory tract infection and hospitalization, especially in children. Highly mutagenic nature and antigenic diversity enable the RSV to successfully survive in human population. We conducted a molecular epidemiological study during 2017–2021 to investigate the prevalence and genetic characteristics of RSV.

**Methods:**

A total of 6499 nasopharyngeal (NP) swabs were collected from hospitalized children at Department of Pediatrics, Guangdong Provincial Hospital of Traditional Chinese Medicine, Guangzhou, Guangdong, China. All NP swab specimens were preliminary screened for common respiratory viruses and then tested for RSV using specific PCR assays. Partial G genes of RSV were amplified for phylogenetic analysis and genetic characterization.

**Results:**

The overall detection rate for common respiratory viruses was 16.12% (1048/6499). Among those, 405 specimens (6.20%, 405/6499) were found positive for RSV. The monthly distribution of RSV and other respiratory viruses was variable, and the highest incidence was recorded in Autumn and Winter. Based on the sequencing of hypervariable region of G gene, 93 RSV sequences were sub-grouped into RSV-A (56, 60.2%) and RSV-B (37, 39.8%). There was no coinfection of RSV-A and RSV-B in the tested samples. Phylogenetic analysis revealed that RSV-A and RSV-B strains belonged to ON1 and BA9 genotypes respectively, indicating predominance of these genotypes in Guangzhou. Several substitutions were observed which may likely change the antigenicity and pathogenicity of RSV. Multiple glycosylation sites were noticed, demonstrating high selection pressure on these genotypes.

**Conclusion:**

This study illustrated useful information about epidemiology, genetic characteristics, and circulating genotypes of RSV in Guangzhou China. Regular monitoring of the circulating strains of RSV in different parts of China could assist in the development of more effective vaccines and preventive measures.

## Background

Respiratory syncytial virus (RSV) is considered a serious respiratory tract infection vulnerable of humans especially infants and children [1, 2]. According to an estimate, approximately 70,000 children died annually and approximately 3.4 million people are admitted in hospitals worldwide due to RSV infections [3], putting a substantial burden on the healthcare system of both high- and low-income countries. During the last decade, RSV infections have become more prominent and apparent, causing illness not only in children but also in elderly people (≥ 65 years). Although treatment options for RSV infection are limited, advances in the understanding of the virus biology and structure have led to the development of the world's first RSV vaccine for individuals aged 60 years and older (RSVPreF3, Arexvy, GSK) leaving a significant clinical [4–6]. RSV mainly spreads through nasal or oral secretions of infected persons and no animal reservoir or intermediate host for RSV has been reported to date. A healthy person gets infected either directly (via large droplets) or indirectly by touching contaminated surfaces such as cribs, toys, phones, doorknobs, and tabletops. RSV only induces partial immunity and reinfections are quite common in children and adults due to virus strain variations and virus evolution [7].

RSV is an enveloped RNA virus of *Pneumoviridae* family of respiratory viruses [8]. It is negative-sense, single-strand virus having a genome length of about 15.2 kb, containing 10 genes that encode 11 proteins including NS1, NS2, N, P, M, SH, G, F, M2-1, M2-2 and L. Based on antigenic and genetic variations, RSV strains can be divided into two major subtypes (RSV-A, RSV-B) [9]. Usually, both subtypes cocirculate simultaneously in the human populations but RSV-A viruses tend to predominate. The main difference between these subtypes is due to remarkable amino acid variations in the attachment (G) glycoprotein which can accommodate drastic changes. The G and F (fusion) are two major virus surface proteins that mediate virus attachment and entry into host cell, respectively. Full length G protein (300 aa) consists of three major domains including an N-terminal cytosolic domain (aa1–36), a transmembrane domain (aa37–67), and an ectodomain (aa68–298). There are two hypervariable mucins like domain (MLD-1, MLD-2) in the ectodomain region of the G protein. There is a conserved central domain (CCD, aa164–176) and a heparin-binding domain (HBD, aa186–224) located in between two MLD. A small cysteine nose involving four cysteines (Cys173, Cys176, Cys182, and Cys186) partially overlaps with CCD. Five amino acids (aa 182–186) constitute a CX3C motif which plays a critical role in RSV infections [10]. There is heavy glycosylation of *N*-linked and *O*-linked sugars in the G protein which makes G gene to high level of amino acid variations among RSV genes. The G protein is an important target for the development of vaccines and anti-viral agents [11]. Sequencing and analysis of second variable region or C-terminal region of the G protein is widely used to study evolution and genotyping of RSV isolates [12, 13]. Based on nucleotide sequence analysis, RSV subtypes can be divided further into several genotypes which have been discovered over the years. To date, researchers have found 15 genotypes for RSV-A (GA1-7, NA1-4, ON1-2, SAA1, and CBA) and 30 genotypes for RSV-B (GB1-4, BA1-14, BAc, SAB1-4, URU1-2, CB1 (GB5), CBB, BA-CCA, BA-CCB, and THB). Variable pathogenicity has been demonstrated by different genotypes of RSV and these can co circulate in the same area and period. However, usually one of the subtypes or genotypes predominate in most of the epidemics [14]. Prototype ON1 (Ontario) genotype was discovered in Ontario, Canada in 2010 which demonstrated a duplication of 72 nucleotides within second variable region of G gene [15]. Since 2010, ON1 genotypes have rapidly spread to many countries [16–20] and notably undergoing evolutionary changes. BA genotype (Buenos Aires) was first identified in Buenos Aires, Argentina in 1999 which showed a duplication of 60 nucleotides in the second variable region of G gene [21, 22]. Since then, the BA genotype has spread worldwide and 12 genotypes of BA have been identified [16, 21, 23, 24].

During the previous five years, RSV has become a dominant respiratory virus in China and was frequently detected among children suffering from respiratory tract infections [10, 16, 20, 25–28]. Recently, several new genotypes of RSV have been confirmed worldwide suggesting a rapid evolutionary process in RSV. Both RSV-A and RSV-B subtypes co-circulate in China. The ON1 genotype was first identified in Shanghai, China in 2011 and since then quickly spread and became the dominant RSV-A genotype in China [16, 20, 25, 26, 29]. Similarly, BA genotypes have been detected in children in different cities of China, indicating their widespread circulation in China. Recently, ON1 and BA9 genotypes are causing huge morbidity among children and have been declared dominant genotypes in China [30]. According to Zhang et al. [29], the RSV-A prevailing season lasts 6 weeks longer than the RSV-B season. Some studies from China have reported a region-to-region variation in the prevalence of RSV [10, 16, 18, 20, 26, 28–30]. There is a scarcity of epidemiological and continuous surveillance data from different parts of China and only limited data have been published on RSV genotypes circulating in China. Therefore, this study was designed to investigate the epidemiology and genotypic characteristics of RSV from hospitalized patients at the Second Affiliated Hospital of Guangzhou University of Chinese Medicine, Guangzhou, Guangdong province, China. As part of infection surveillance program, we collected specimens from patients from September 2017 to December 2021. Besides RSV detection, other common respiratory viruses were also detected using commercial assays in this study.

## Methods

### Specimen collection

**Fig. 1:**
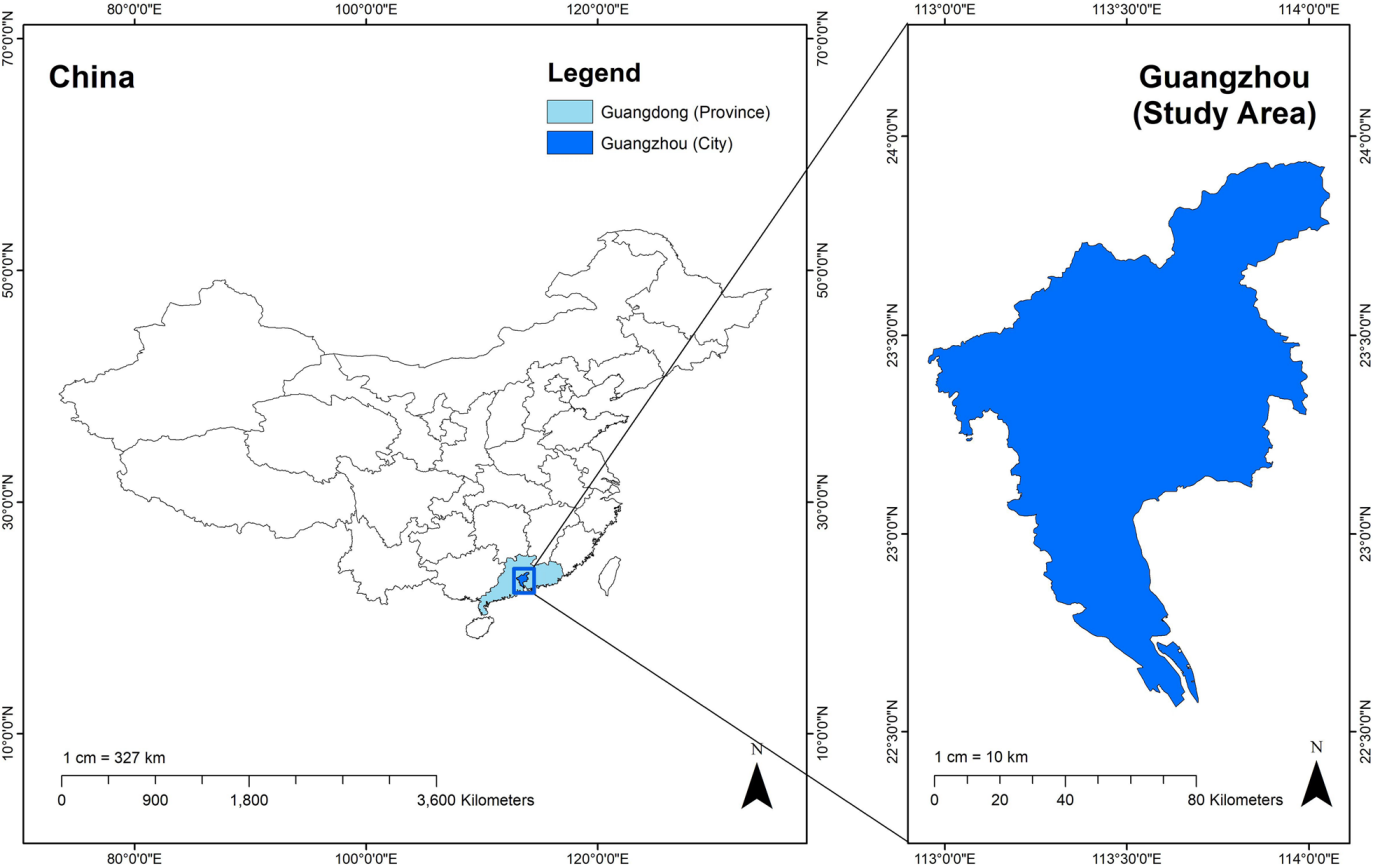
Map showing sampling area in China (Guangzhou). Nasopharyngeal (NP) swabs were collected from patients suffering from respiratory infections from September 2017 to December 2021 at Department of Pediatrics, Guangdong Provincial Hospital of Traditional Chinese Medicine, Yuexiu District, Guangzhou, Guangdong, China

All samples were collected with the consent of patients. This study was approved by the Ethics and Research Council of The Second Affiliated Hospital of Guangzhou University of Chinese Medicine, Guangzhou, China (Approval number: ZE2020-3034-01). A total of 6499 nasopharyngeal (NP) swabs were collected from children (≤ 14 years of age) suffering from respiratory infections from September 2017 to December 2021 at Department of Pediatrics, The Second Affiliated Hospital of Guangzhou University of Chinese Medicine, Guangzhou, Guangdong, China (Fig. 1). Patients who were showing clinical signs of fever (> 37.5 °C), cough, runny nose, sputum, dyspnea, and sore throat were included in this study. Clinical information, demographic data and laboratory testing for each patient were conducted by hospital staff and physicians (Table 1). The NP swab specimens were then transported immediately on wet ice to the hospital laboratory for testing and analysis. After testing, all specimens were stored at − 80 °C for further retesting.

**Table 1:** Demographic characteristics and clinical symptoms associated with hospitalized children (n = 93) suffering from RSV infection

	Total number of specimens positive for RSV (n = 405)	Randomly selected specimens for RSV subtyping (n = 93)		RSV subtypes			
				RSVB		RSVA	
	n	n	%	n	%	n	%
Age group (years)							
< 1	237 (58.5%)	29	31.1	19	65.5	10	34.4
1–4	132 (32.5%)	62	66.6	36	58.9	26	41.9
5–14	36 (8.9%)	2	2.1	1	50	1	50
Gender							
Male	217 (53.6%)	60	64.5	35	58.3	25	41.6
Female	188 (46.4%)	33	35.4	21	63.6	12	36.3
Clinical characteristics							
Cough	388 (95.8%)	89	95.6	54	60.6	35	39.3
Sneezing	184 (45.4%)	4	4.3	2	50	2	50
Nasal discharge	355 (87.6%)	66	70.9	41	62.1	25	37.8
Malaise	207 (51%)	2	2.1	2	100	0	0
Sore throat	178 (43.9%)	3	3.2	2	66.6	1	33.3
Myalgia	169 (41.7%)	2	2.1	2	100	0	0
Fever	405 (100%)	85	91.3	53	62.3	32	37.6

### Preliminary screening of NP specimens for common respiratory viruses

All NP swab specimens were first screened for the presence of seven common respiratory viruses including RSV, influenza A (IFA), influenza B (IFB), adenovirus (AdV), parainfluenza 1 (PIV1), parainfluenza 2 (PIV2) and parainfluenza 3 (PIV3) by using a commercially available immunofluorescence D3 Ultra DFA Respiratory Virus Screening and Identification Kit (Quidel, CA, USA). This kit utilizes monoclonal antibodies and immunofluorescence technology to detect viral antigens from NP swab specimens collected from patients suffering from respiratory tract infections. Based on our previous study, this screening kit provides specificity and reliability in testing common respiratory viruses [31].

### Nucleic acid extraction and reverse transcription

Viral nucleic acid (RNA) was extracted from immunofluorescence assay RSV positive specimens (n = 405) using a TIANamp Virus nucleic acid extraction Kit (TIANGEN, Beijing, China). According to the manufacturer’s instructions, complementary DNA (cDNA) strands were synthesized from RNA templates by using SuperScript III Reverse Transcription Kit (Thermo Scientific, USA). Later, cDNA specimens were processed for RSV detection and sequencing using polymerase chain reaction (PCR) assay.

### G gene sequencing of RSV-A and RSV-B

A nested PCR was performed on specimens to amplify second variable region of G gene of RSV by using a C1000 Touch™ Thermal Cycler (Bio-Rad Laboratories, Inc, Hercules, California, US) and RT-PCR kit (TaKaRa, Dalian, China). The first round of PCR amplification was performed by using a pair of forward and reverse primers (AG20-5-GGGGCAAATGCAAACATGTCC-3 and F164 ′5-GTTATGACACTGGTATACCAACC-3′). These primers targeted and amplified the second variable region of the G gene and part of the F gene (approximately 1220 bp). For the second round of PCR amplification, we used another pair of reverse and forward primers (BG10-5-GCAATGATAATCTCAACCTC-3 and F1-5-CAACTCCATTGTTATTTGCC-3).We used following thermocycling conditions for both round of amplifications: 94 °C for 5 min, followed by 40 cycles at 94 °C for 30 s, 54 °C for 30 s, and 72 °C for 1 min, and a 5 min final extension at 72 °C as described elsewhere [20]. PCR amplicons from second round of amplification were analyzed on 1% agarose gels and approximately 844–901 bp of PCR amplicons were observed by using GenoSens 1880 gel imaging analysis system (GenoSens 1880, Clinx, Shanghai, China). Among 405 RSV positive amplicons, 93 were selected randomly, processed, and sent for sanger sequencing to Guangzhou Huayin Medical Laboratory Center Co., Ltd., Guangzhou, 510663 Guangdong, China. Sanger sequencing of G gene was performed by using the BigDye Terminator 3.1 kit and ABI-PRISM 3730XL DNA sequencer (Applied Biosystems). These sequences were deposited in the GeneBank (NCBI Accession numbers OR456347-OR456439).

### Nucleotide sequence analysis

Raw nucleotide sequences were analyzed, edited, and aligned, for phylogeny using BioEdit Software version 7.2. Sequence electropherograms were analyzed carefully and nucleotide ambiguities were excluded. To make sequencing data more reliable, we aligned forward and reverse sequences together to generate a consensus sequence. Multiple sequences were assembled and aligned using Clustal W version 2.0. Reference sequences for the G gene of subgroup A and B were retrieved from GenBank (http://www.ncbi.nlm.nih.gov) as of December 2022. All sequences were trimmed and aligned according to the second variable region of G gene. Molecular Evolutionary Genetic Analysis (MEGA version 11.0)® was used to construct and analyse phylogeny tree [32]. Phylogenetic trees were constructed using maximum-likelihood method with the Kimura 2-parameter model. BioEdit Software version 7.2 was used for deduced amino acid sequences analysis and comparison with references strains from GenBank to identify amino acid substitutions, deletions, and insertions in the G protein. We used NetNGlyc 1.0 server (http://www.cbs.dtu.dk/services/NetNGlyc/) and the NetOGlyc 4.0 server (http://www.cbs.dtu.dk/services/NetOGlyc/) to predict potential *N*-linked and *O*-linked glycosylation sites in the second variable region of the G protein respectively [33, 34]. A universal rule was adapted to characterize glycosylation sites. According to that rule, presence of asparagine (N)-X-Serine (S)/Threonine (T) [where X is any amino acid except Proline (P)] indicates a potential *N* linked glycosylation site while presence of Serine (S) and threonine (T) amino acid residues are considered potential *O* linked glycosylation sites.

### Statistical analysis

Excel 2010 (Microsoft Co., Washington, DC, USA) was used for data processing, and SPSS (v18.0, SPSS, Chicago, IL, USA) was used for statistical analysis. The demographic and epidemiological data were analyzed for statistical significance using the Chi-square test or Fisher’s exact test as appropriate. Kruskal–Wallis test was utilized for comparisons between two or more groups. A value of *p* < 0.05 was considered as statistically significant.

## Results

### Detection of respiratory viruses

We have described detection rates of several respiratory viruses from September 2017 to December 2021 in this study. A total of 6499 specimens were tested through Immunofluorescence assay for preliminary screening of respiratory viruses. Overall detection rate for common respiratory viruses from total collected specimens was 16.12% (1048 /6499). Among those, 405 specimens (6.20%, 405/6499) were found positive for RSV. Among respiratory viruses, the highest detection rate was of RSV (6.20%, 405/6499) followed by IFA 2.8% (180/ 6499), AdV 2.13% (139/6499), PIV3 1.9% (123/1048), IFB 1.6% (101/6499) PIV1 1.3% (83/6499) and PIV2 (0.3%) (19/6499) as shown in Table 1. Among total positive specimens (n = 1048), detection rate for RSV, IFA, AdV, PIV3, IFB and PIV2 was 38.45%,17.17%, 13.26%, 11.74%, 9.64%, 7.92% and 1.81% respectively (Table 2). Out of 405 RSV positive specimens, 93 specimens were selected for RSV subtyping, genotyping, and phylogenetic analysis. Surprisingly, all RSV positive samples were found negative for coinfection with IFA, IFB, AdV, PIV1, PIV2 and PIV3.

**Table 2:** Detection rate of respiratory viruses among total specimens collected in different years (2017–2021)

Year	Total specimens	Detection rate for seven respiratory viruses among total specimens (Total number of positive specimens for each virus/ Total number of collected specimens)						
		IFB	RSV	AdV	IFA	PIV1	PIV2	PIV3
2017	602	11 (1.82%)	29 (4.8%)	5 (0.83%)	0 (0.00%)	7 (1.2%)	1 (0.2%)	8 (1.32%)
2018	2053	32 (1.56%)	119 (5.8%)	52 (2.53%)	52 (2.53%)	28 (1.4%)	4 (0.2%)	34 (0.15%)
2019	1926	44 (2.28%)	103 (5.3%)	73 (3.8%)	101 (25.63%)	12 (0.62%)	13 (0.7%)	48 (2.5%)
2020	716	3 (0.42%)	80 (11.17%)	6 (0.84%)	26 (5.24%)	30 (4.2%)	0 (0.00%)	15 (2.09%)
2021	1202	11 (0.92%)	72 (6%)	3 (0.25%)	1 (0.083%)	6 (0.5%)	1 (0.083%)	18 (1.5%)
Total	6499	101 (1.6%)	403 (6.20%)	139 (2.13%)	180 (2.8%)	83 (1.3%)	19 (0.3%)	123 (1.9%)

*IFA* Influenza A virus, *IFB* influenza B virus, *PIV1* parainfluenza virus type 1, *PIV2* parainfluenza virus type 2, *PIV3* parainfluenza virus type 3, *AdV* adenovirus, *RSV* respiratory syncytial virus.

### Demographic and clinical characteristics of RSV positive cases

The 405 RSV positive patients identified in this study included 217 (53.6%) males and 188 (46.4%) females. RSV positivity between males and females were found non-significant (*p* > 0.05). The RSV positive patients were distributed across age groups (age groups < 1, 1–4, and 5–14 years). Distribution of RSV detection rate among the different age groups was significant different (*p* < 0.05). The highest prevalence of RSV infections was found among children who were below 1 year of age (58.5%), followed by those between 1–4 years (32.5%) and 5–14 years of age (8.9%). Our preliminary analysis revealed higher incidence of RSV in children especially under 1 year of age, mostly showing clinical signs of fever and cough (Table 1). The median duration of hospital stay was 7 days for children suffering from RSV infections in this study. The most prominent clinical characteristics included fever (100%), cough (95.8%) and nasal discharge (87.5%) (*p* < 0.05). However, we did not find meaningful differences in age groups and clinical characteristics between RSV-A and RSV-B positive patient and their association was non-significant (*p* > 0.05).

### Monthly distribution of respiratory viruses

**Fig. 2:**
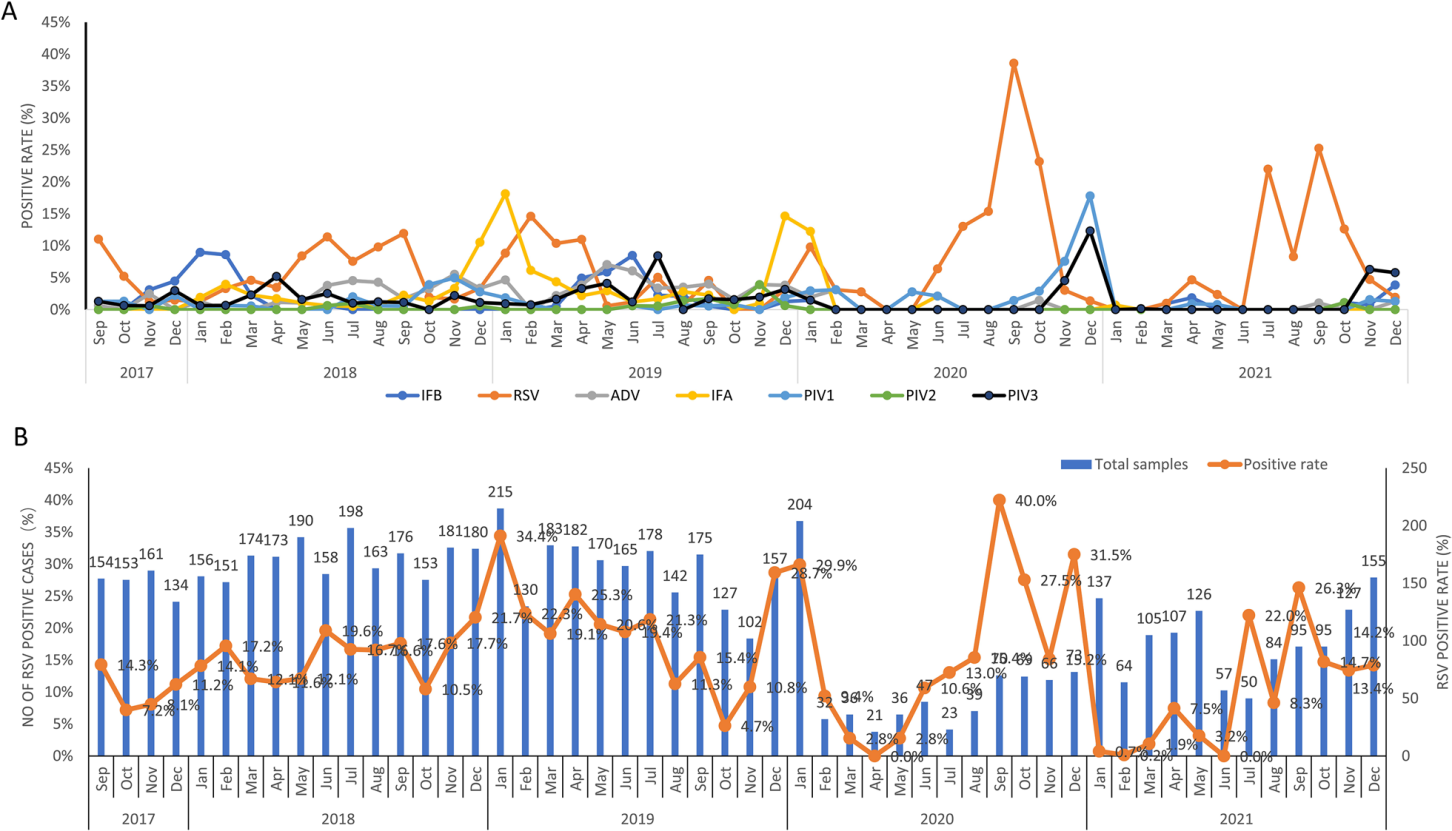
(**A**) Monthly positive rate of different respiratory viruses from September 2017 to December 2021. IFA, Influenza A virus IFB, influenza B virus; PIV1, parainfluenza virus type 1; PIV2, parainfluenza virus type 2; PIV3, parainfluenza virus type 3; AdV, adenovirus; RSV, respiratory syncytial virus. (**B**) Monthly distribution of respiratory syncytial virus (RSV) from September 2017 to December 2021

We collected and analyzed data for monthly distribution of RSV and other respiratory virus cases as described previously [35]. There were statistical associations between respiratory viruses (IFA, IFB, AdV, RSV, PIV1, PIV2 and PIV3) and months (*p* < 0.05 or *p* > 0.05). Monthly distribution of RSV and other respiratory virus positive cases has been presented in Fig. 2. The annual RSV incidences were 4.8% (29/602), 5.8% (119/2053), 5.3% (103/1926), 11.17% (80/716), and 6% (72/1202) from 2017 to 2021, respectively (Table 2). Notably, we found significantly higher RSV incidences in the year 2020 (11.17%, *p* < 0.05). Non-significantly higher number of RSV cases were observed in January 2018, January 2019, January 2020, September 2020, December 2020, and September 2021 (*p* > 0.05).

### Sequence alignments and phylogenetic analysis

**Fig. 3:**
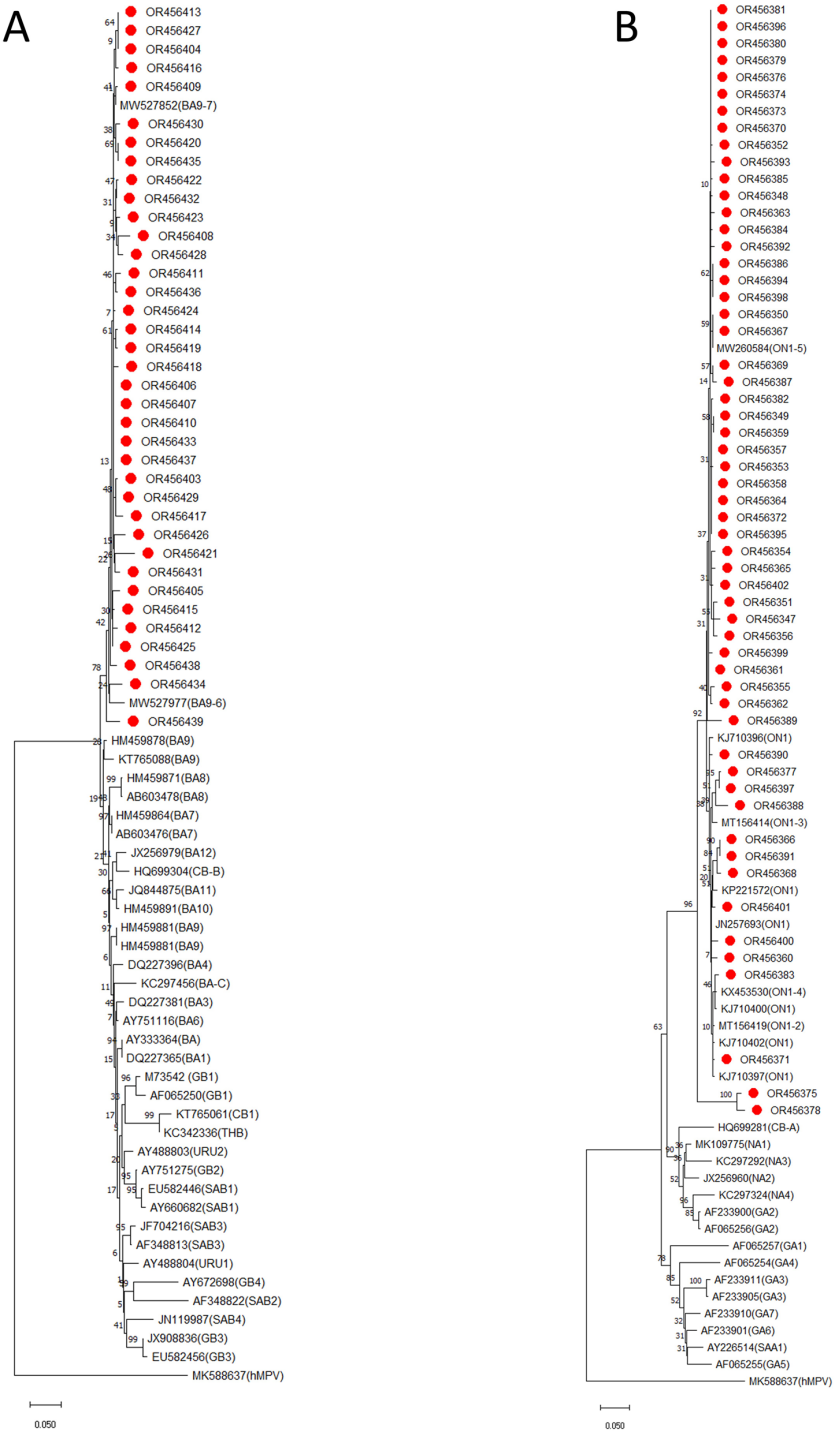
Phylogenetic tree was constructed using the Neighbor-Joining method with the bootstrap test (1000 replicates) using MEGA 11 software. The evolutionary distances were computed using the p-distance method and are in the units of the number of base differences per site. (**A**) Phylogenetic analysis of G gene sequences of RSV-A strains circulating in Shanghai, September 2017–December 2021. The present study RSVA strains are indicated by ‘‘red circles’’ followed by their NCBI accession numbers. RSV-A reference strains representing known genotypes were retrieved from GenBank and included in the tree (labels include accession number). Human metapneumovirus (MK588637) was used as an outgroup. The scale bar represents the number of nucleotides substitutions per site. (**B**) Phylogenetic analysis of G gene sequences of RSV-B strains circulating in Shanghai, September 2017–December 2021. The present study RSV-B strains are indicated by ‘‘red circles’’ followed by their NCBI accession numbers. RSV-B reference strains representing known genotypes were retrieved from GenBank and included in the tree (labels include accession number). Human metapneumovirus (MK588637) was used as an outgroup. The scale bar represents the number of nucleotides substitutions per site

All PCR amplicons (n = 93) were successfully sequenced and yielded good quality sequences for further analysis. All sequences were cleaned, edited, and aligned with representative reference genotypes. Among 93 RSV sequences, 56 (60.2%) and 37 (39.8%) were categorized into RSV-A and RSV-B respectively. There was no coinfection of RSV-A and RSV-B in tested samples. Sequence and phylogenetic analysis of G gene showed that RSV-A strains (56, 60.2%) belonged to ON1 genotype with the mean nucleotide sequence homology of 99%. They all aligned closely to a novel ON1 genotype which was first identified in 2010 in Ontario, Canada (Fig. 3a). There was 96.4% sequence homology nucleotide level and 93.6% sequence homology at amino acid level between our study RSV-A strain (ON1) sequence and ON1 reference strain (Canada strain, ON67-1210, GenBank number: JN257693).All 56 RSV ON1 genotype were closely related to ON1 strain (MW455132.1, MN007037.1) previously isolated in Guangdong province ( percent identity 98–99%).

All RSV-B strains (n = 37, 39.8%) belonged to BA genotype and clustered with strains that were previously assigned to the BA-9 genotype with a 60-nucleotide duplication as shown in Fig. 3b. A sequence homology of 94.8–97.2% at the nucleotide level and 89–94% at the amino acid level was observed among the sequences of RSV-B and the BA reference strain (AY333364) was. All 37 RSV-B strains of our study belonged to the BA9 genotype and were close to Guangzhou strains previously reported in China (MW527906). These findings indicate that ON1 and BA9 are the dominant genotypes in Guangzhou from 2017 to 2021 (Table 3).

**Table 3:** Detection rate of respiratory syncytial virus (RSV) among positive specimens in different years (2017–2021)

Year	Total specimens	Total positive rate for respiratory viruses	Detection rate for seven respiratory viruses among positive specimens (Total number of positive specimens for each virus/ Total number of positive specimens)						
			IFB	RSV	AdV	IFA	PIV1	PIV2	PIV3
2017	602	61 (9.8%)	11 (18.03%)	29 (47.54%)	5 (8.19%)	0 (0.00%)	7 (11.47%)	1 (1.64%)	8 (13.11%)
2018	2053	321 (15.64%)	32 (9.97%)	119 (37.07%)	52 (16.19%)	52 (16.19%)	28 (8.72%)	4 (1.25%)	34 (10.59%)
2019	1926	394 (20.46%)	44 (11.17%)	103 (26.14%)	73 (18.53%)	101 (25.63%)	12 (3.05%)	13 (3.30%)	48 (12.18%)
2020	716	160 (22.35%)	3 (1.87%)	80 (50%)	6 (3.75%)	26 (16.25%)	30 (18.75%)	0 (0.00%)	15 (9.37%)
2021	1202	112 (9.32%)	11 (9.82%)	72 (64.28%)	3 (2.67%)	1 (0.89%)	6 (5.36%)	1 (0.89%)	18 (16.07%)
Total	6499	1048 (16.12%)	101 (9.64%)	403 (38.45%)	139 (13.26%)	180 (17.17%)	83 (7.92%)	19 (1.81%)	123 (11.74%)

*IFA* Influenza A virus IFB, influenza B virus, *PIV1* parainfluenza virus type 1, *PIV2* parainfluenza virus type 2, *PIV3* parainfluenza virus type 3, *AdV* adenovirus, *RSV* respiratory syncytial virus

### Deduced amino acid sequence analysis

**Fig. 4:**
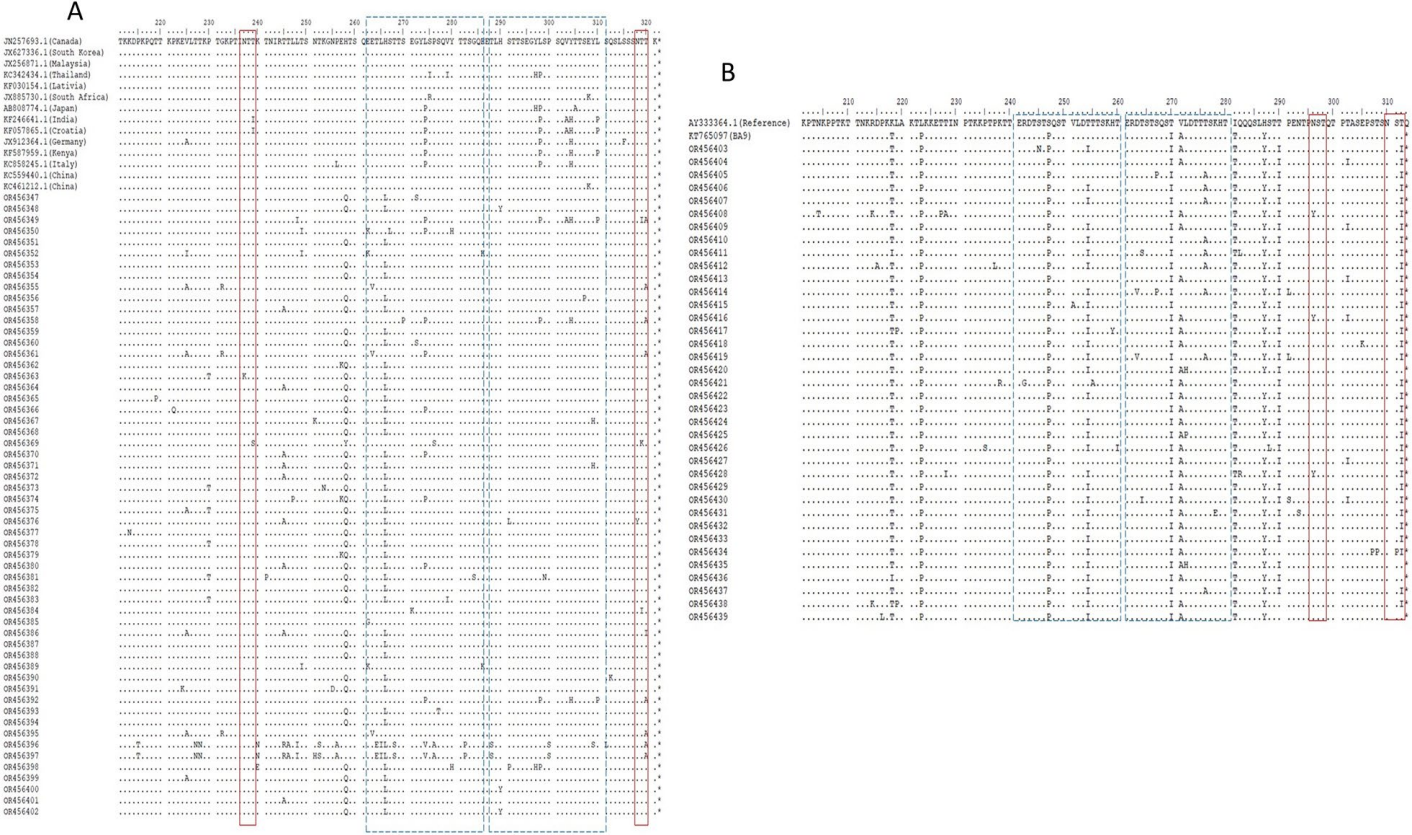
(**A**) Alignment of amino acid sequences of RSV-A strains in the second variable region of G protein. Alignments are shown relative to the sequence of ON1 strain first described in Canada (GenBank accession number JN257693). Alignment of sequences was performed using the ClustalW1.6 method via BioEdit software. The amino acid positions correspond to positions 210 to 322 of the G protein of the ON1 strain. Identical residues are indicated by dots, asterisks indicate the position of stop codons. Dashed line boxes frame the 23 amino acid duplicated region of the 24 amino acid insertion. Solid line boxes highlight predicted N-glycosylation sites. This study RSVA strains are labeled with their NCBI accession numbers. (**B**) Alignment of amino acid sequences of RSV-B strains in the second variable region of G protein. Alignments are shown relative to the sequence of a prototype BA strain (GenBank accession number AY333364). Alignment of sequences was performed using the ClustalW1.6 method via BioEdit software. The amino acid positions correspond to positions 201 to 313 of the G protein of the BA strain. Identical residues are indicated by dots, asterisks indicate the position of stop codons. Dashed line boxes frame the 20 amino acid duplication and insertion. Solid line box highlights predicted N-glycosylation sites. This study RSV-B strains are labeled with their NCBI accession numbers

We aligned and compared RSV-A strains (ON1 genotype) of our study with reference ON1 strain originated from Canada (Canada strain, ON67-1210, GenBank number: JN257693) and RSV-A prototype strain A2. An insertion of 24 amino acids (72 nucleotides) lengthened the G protein when compared to RSV-A prototype strain A2. We identified several amino acid substitutions at MLD1 and MLD2 of the G protein when compared to the prototype ON1 strain (N257693). Five amino acid substitutions (T113I, V131D, N178 G, H258Q and H266 L) were notable in most of ON1 strains belonging to cluster 1 (ON1-5 clade). Two substitutions (N178G, H266L) were considered significantly important as they were present close to CX3C motif in CCD (N178G) and within antigenic site (265–273aa) indicating continuous evolution and adaptation of ON1 lineages in China which may play a role in RSV transmission and disease severity. A total of 228 amino acid changes were noticed at 43 different sites in the 56 sequences of second variable region of the G protein (aa210 to aa321) of genotype ON1 compared with the reference ON1 genotype of RSV-A. The most amino acid variations were shown by two RSV-A strains of this study (OR456396, OR456397) which indicated variation in twenty-one and twenty amino acids respectively when compared to reference strain. Out of 43 sites, only 7 sites showed more than 2% frequency of amino acid change [V225A (3.5%), P230T (2.6%), T245A (4.4%), H258Q (18%), H266L (19%), L274P (3%), T320A (3%)]. While other 36 sites showed less than 1% frequency of amino acid change. Amino acid position 258 (H258Q, 21.31%), and 266 (H266L, 22.33%) showed the highest amino acid changes (Fig. 4a). The analysis of potential N-glycosylation sites revealed two potential N glycosylation sites at amino acid positions 237 and 318 in second variable region of the G protein of ON1 strains. However, N glycosylation was lost in two ON1 strains (OR456363, OR456369) due to substitutions at N237K and T239S, respectively.

Similarly, eight ON1 strains (OR456349, OR456355, OR456358, OR456361, OR456392, OR456395, OR456396, OR456397) lost N glycosylation due to T320A substitutions. Analysis of the second variable region predicted different pattern of O-glycosylation sites with 35–46 potential O-glycosylation sites among genotype ON1 sequences when compared to the Ontario reference strain. The amino acid positions most likely to have O-glycosylation in ON1 strains of present study are T211, T219, T220, T220, P222, L226, T227, T228, T231, P234, T235, T238, T239, T241, R244, T245, T246, T249, S250, T252, K253, T259, S260, T264, S267, T268, T269, S270, S275, P276, S277, T281, T282, S283, S299, S301, T305, T306, S307, S311, S313, S315, S316, S317, T319, T320 (refer to OR456347).

We also aligned and compared the second variable region of protein G of RSV-B strains (BA9 genotype) of our study with their respective reference sequences BA (AY333364). There were 396 amino acid substitutions in all 37 sequences of second variable region of the G protein of RSV-B genotype (BA9), compared with BA reference sequences (AY333364). The amino acid variations analyses revealed 10 sites [K218T (9.3%), L223P (9.3%), S247P (9.3%), T254I (7.6%), T270I (9.3%), V271A (6.8%), T281I (9.3%), H287Y (8.8%), T290I (8.6%), T312I (8.3%)] with more than 5% frequency in amino acid changes and 102 sites with less than 5% frequency of amino acid changes in second variable region of the G protein (aa200 to aa312) (Fig. 4b).

Interestingly, we observed several differences in amino acid frequency at position 254, 275, 290. 302 and 312 when compared reference strain of BA9 from China (KT765097, VRL-2016). Deduced amino acid analysis revealed two *N*-glycosylation sites at 296 and 310 amino acid positions of genotype BA9 sequences. However, these *N*-glycosylation sites at position 310 were missing in most of the BA9 strains due to due toT312I substitution and only four strains (OR456439, OR456436, OR456435, OR456420) showed potential *N*-glycosylation sites at residue 310. Similarly, three BA9 strains (OR456416, OR456408, OR456428) lost potential *N*-glycosylation sites due to N296Y substitution. Analysis of the second variable region predicted different patterns of *O*-glycosylation with 27–50 potential *O*-glycosylation sites among genotype BA9 sequences when compared to the BA9 reference strain (KT765097). The amino acid positions most likely to have *O*-glycosylation in BA9 strains of present study are T211, P216, K218, T218, T222, T227, T228, I229, P231, T232, T236, T239, T240, R242, T244, S245, T246, S249, T250, T254, T255, T256, S257, T260,T264, S265, T266, S267, S269, T274,T275, T276, S277, T280, I281, S285, S288, T289, T290, T294, S297, T298, T300, T302, S304,S307,T308, S309, S311 and T312 for genotype BA9 (refer to OR456403).

## Discussion

Respiratory viruses usually cause high morbidity and mortality with acute respiratory infections, especially in children. Viral infections involving respiratory tract get out of hand quickly via sneezing and coughing in contrast to other type of infections. For better therapeutic and preventive measures, it is imperative to understand the epidemiology and genotypic characteristics of respiratory viruses. RSV is a key respiratory virus worldwide and imposes a serious burden on health care settings by causing severe morbidity and hospitalization in children. Knowledge of circulating RSV genotypes can help us to better understand RSV infection and to implement suitable preventive measures [5, 10, 16, 20, 25, 29].

In our study, we collected and analyzed NP swab specimens during September 2017–December 2021 to understand the genetic diversity and patterns of RSV subtypes in Guangzhou, China. NP swab specimens are widely adopted by researchers for the surveillance of respiratory viruses including RSV [36–38]. Overall detection rate for all respiratory viruses from total collected specimens was 16.12% (1048 /6499). RSV and IFA were most frequently detected during 2017–2021. These findings are consistent with previous reports from China [38, 39]. Detection rate of AdV was third highest in this study. AdV is considered an important cause of respiratory tract infections and mainly infect preadolescent (2–12 years old) [31, 39].Common distribution of these respiratory viruses in children in China warrants careful clinical diagnosis and treatment. During our study, we found NP swab specimens to be the most suitable and reliable for respiratory virus detection as these were easy to collect and yielded good quality nucleic acid for molecular assays. These findings are consistent with previous reports in literature [37, 40]. A total of 405 specimens (6.20%, 405/6499) were found positive for RSV. Among 93 RSV sequences, 56 (60.2%) were sub-grouped as RSV-A and 37 (39.8%) as RSV-B. Surprisingly low detection rate of RSV could be associated with SARS-CoV2 pandemic precautionary measures and strict lockdown in China. Notably, we did not find any coinfection between RSV-A and RSV-B subtypes in tested samples. In past, some conflicting and non-conclusive reports have been published regarding this trend of RSV infections [19, 25]. As expected, we noticed highest detection rate of RSV among infants (< 1 year of age). It has been established previously that RSV infections are common in children less than 1 years of age and with increasing age, chances of getting RSV infections becomes low [5, 29, 39]. This indicates that RSV has high pathogenicity for children possibly due to immature immune system. In our study, most of the children with fever and cough were presented at hospital clinic for treatment. These findings are consistent with other similar studies [29, 37, 41, 42]. In RSV positive patients, we found a significant longer duration of clinical symptoms (*p* < 0.05) when compared to RSV negative patients. In our study, we found 1048 specimens positive for respiratory viruses (16.12%, 1048 /6499) indicating the role of these respiratory viruses in causing respiratory diseases. These findings align with previously published reports [26, 37, 38, 42]. Surprisingly, we did not observe any co-infection between RSV and other respiratory viruses in this study. This finding is inconsistent with several studies in China and other countries where coinfection of RSV with other respiratory viruses was common in patients suffering from respiratory tract infections [25, 29]. Recently, Chen et al. [25] reported high rate of coinfection between RSV and other notable respiratory viruses. The impact of these coinfections is still non conclusive and controversial. According to some studies, coinfections can exacerbate the clinical signs while others reported no effect on diseases severity and clinical signs [25, 43, 44]. Therefore, further study about viral coinfection is needed. Furthermore, we did not screen RSV positive samples for bacterial coinfections. Zhang et al. [29] reported significant detection of *Streptococcus pneumoniae, Hemophilus influenzae*, mycoplasma and chlamydia in patients suffering from RSV infections.

Although, RSV positive cases were observed throughout the year. However, highest incidence of RSV infections was recorded in Autumn and Winter season during our study. These findings are consistent with reports from other regions in China [26, 29, 37–39, 41] who reported peak RSV incidences during the winter and early spring months. However, these findings conflict with some studies which reported an association between a region and seasonal prevalence of the RSV [45, 46] and indeed seasonal cases of RSV vary among different countries owing to different climate and seasons. Phylogeny indicated that all RSV-A strains (n = 56) aligned closely with a novel ON1 genotype which was first identified in 2010 in Ontario, Canada [15]. Since 2010, ON1 genotype RSV-A has been reported in several countries of the world including China, Japan, South Korea, India, Malaysia, Germany, Latavia, Cyprus, Kenya, Thailand, and other countries [17, 27]. In China, the first case of ON1 genotype was detected in Shanghai in 2011 and since then this genotype spread to all parts of China and become the predominant genotype circulating in China by replacing NA1 genotypes which were considered the main genotypes before 2013 in China [10, 27].Tsukagoshi et al. [47] reported that ON1 genotypes have evolved from NA1 genotypes and according phylogenetic analysis estimation ON1 genotypes emerged around 2008–2010 [40, 48, 49]. Between 2003 and 2008, GA2 was the predominant genotype in China which was replaced by NA1 genotype from 2008 to 2013 [10, 27, 50]. During this study, we found ON1 as predominant genotype confirming rapid spread of this genotype This finding is consistent with recent studies in China, reporting dominance of ON1 genotype [10, 16, 20, 25–27, 39]. To date, ON1 genotype has been detected in 21 countries indicating widespread dissemination of this genotype. These RSV genotypes have the potential to evolve and gaining some fitness advantages than other genotypes [10, 17, 27]. It is expected that new lineages could emerge in near future possibly due to antigenic variations and large host population. Recently, Zhao et al. [10] has reported a new ON1 lineage (ON1-5) in Shanghai. Similarly, several nucleotide and amino acid variations were observed in the RSV-A strain sequences indicating RSV is acquiring variations in its G gene at a rapid rate. Similar to previous reports we also found sequence duplication in the second variable region of the G protein. This region contains important antigenic epitopes and can generate new variants especially under immune pressure after natural infections [27]. In addition, sequence duplication in the second variable region of the G protein increases the length and modifies the structure of the G protein leading to enhanced attachment and immune evasion [10, 16].

Phylogenetic analysis of 37 sequences of RSV-B strains revealed BA genotype with a 60-nucleotide duplication, first described by Trento et al. [22] in Buenos Aires, Argentina in 1999 and further differentiated into the genotypes BA9 (n = 37, 100%). Interestingly, nucleotide insertion into BA genotypes were reported many years ago before the discovery of ON1 genotypes. Since the discovery of BA genotypes in 1999, they have circulated worldwide and evolved into 12 genotypes [18, 21, 30]. It is suggested that insertion of nucleotide into genome of BA genotypes gave rise to some fitness advantages for RSV for better attachment and pathogenicity [30].

The lengthening of the G protein genotypes ON1 and BA9 due to duplication of 72 and 60 nucleotide respectively has played a key in rapid spread of these genotypes. It is hypothesized that these changes have provided some fitness and evolutionary advantages, leading to the quick spread and dominance worldwide than other genotypes [25]. The G protein plays a critical role in virus entry into the host cells and its CX3C motif interacts with the human chemokine receptor CX3CR1 to induce infection. Multiple amino acid substitutions in the G protein help virus to evade human immune responses. These properties of the G protein make it a significant candidate for future vaccines development against RSV [51]. In addition, changes in the G protein can help to trace the origin of infection indicating the importance of amino acid substitution identification for the better understanding of circulating genotypes [25].

Deduced amino acid analysis revealed 228 amino acid changes at 43 different sites in the 56 sequences of second variable region of the G protein (amino acid 210-321) of genotype ON1 compared with the reference ON1 genotype of RSV-A. Amino acid position 258 (H258Q, 21.31%), and 266 (H266L, 22.33%) showed the highest amino acid changes indicating virus is under great selection pressure.

There were 396 amino acid substitutions in all 37 sequences of second variable region of the G protein of RSV-B genotype (BA9), compared with BA reference sequences (AY333364). The amino acid variations analyses revealed 10 sites [K218T (9.3%), L223P (9.3%), S247P (9.3%), T254I (7.6%), T270I (9.3%), V271A (6.8%), T281I (9.3%), H287Y (8.8%), T290I (8.6%), T312I (8.3%)] with more than 5% frequency in amino acid changes and 102 sites with less than 5% frequency of amino acid changes in second variable region of the G protein (aa200 to aa312). These findings are consistent with previously published reports in China [10, 25–27].

*N* and *O*-linked glycans on the G protein could play a critical role for the virus to evade host immune responses. It has been established that N and O linked glycosylation in the G protein can alter the attachment and antigenicity properties of RSV [52]. The analysis of potential *N*-glycosylation sites revealed two potential *N* glycosylation sites at amino acid positions of 237 and 318 in second variable region of the G protein of ON1 strains. Analysis of the second variable region of the G protein predicted different pattern of *O*-glycosylation sites with 35–46 potential *O*-glycosylation sites among genotype ON1 Sequences when compared to the Ontario reference strain. Similarly, deduced amino acid analysis revealed two *N*-glycosylation sites at 296 and 310 amino acid positions of genotype BA9 sequences. Analysis of the second variable region of the G protein predicted different pattern of *O*-glycosylation with 16–27 potential *O*-glycosylation sites among genotype BA9 sequences when compared to the BA9 reference strain (KT765097). These mutations indicated a high selection pressure as described previously [26]. These variations in *N* linked glycosylation and *O* linked glycosylation may change the antigenicity and virus fitness of RSV and could potentially lead to more serious respiratory infections in people.

Our study did not reveal any association among specific RSV types, genotypes, lineages, and diseases severity. A great variation in clinical and laboratory parameters were observed among patients. There is conflicting data regarding virulence of RSV and disease severity [7, 53].

Our study had some limitations. First, our study only included patients who visited hospital for treatment, therefore cannot draw a conclusion for the overall RSV infection in a community and may not represent regional epidemic pattern. In addition, several factors such as usage of antibiotic or antivirals prior to visiting hospital for treatment or failure in collection of consecutive samples in holidays may affect outcome of the study. Usually, number of patients visiting hospital for treatment become low during spring festival holidays that could possibly lead to failure in consecutive sample collection. Chinese Lunar year occurs every year in between January and February and most of the people go home to visit their families during these holidays. Second, we could not perform sequencing analysis for specimens which were collected in 2021 due to limited time and funding. Third, we performed genotyping of RSV on second variable region of G gene and could not utilize whole gene sequencing for genotyping. Furthermore, we did not include data about bacterial coinfections, which might also influence disease severity. Coinfection with respiratory viruses and bacteria have been commonly observed in the past [26, 40]. Zhang et al. [29] reported higher bacterial coinfection rate in patients suffering from RSV infections.

## Conclusion

In summary, detection rate of RSV was statistically associated with age and season. Therefore, appropriate knowledge and understanding about RSV infections is highly desired to implement better preventive and therapeutic strategies. Molecular characterization of RSV in Guangzhou China confirmed the co circulation of ON1 and BA9 genotypes of RSV. Deduced amino acid sequence analysis revealed several substitutions which may likely change antigenicity and pathogenicity of RSV. Multiple glycosylation sites were observed in the hypervariable region of the G protein, demonstrating high selection pressure on these genotypes. Due to emergence of several lineages of ON1 and BA9 genotypes, it is quite possible that new lineages will emerge in near future due to rapid evolution in RSV. Continuous and long-term surveillance programs coupled with clinical data must be initiated in Guangzhou to better understand the pattern of seasonal distribution of circulating genotypes of RSV and find any association between emerging genotypes and disease severity.

## Data Availability

Data sharing is not applicable to this article.
